# The Protective Effects of Melatonin on Hainan Black Goats Under Heat Stress: Understanding Its Actions and Mechanisms

**DOI:** 10.3390/antiox14010044

**Published:** 2025-01-03

**Authors:** Hao Wu, Baochun Qin, Guang Yang, Pengyun Ji, Yu Gao, Lu Zhang, Bingyuan Wang, Guoshi Liu

**Affiliations:** 1 Sany Institute of China Agricultural University, Sanya 572025, China; bh2022013@cau.edu.cn (H.W.); sy20223040890@cau.edu.cn (B.Q.); b20223040393@cau.edu.cn (G.Y.); 2 National Engineering Laboratory for Animal Breeding, Key Laboratory of Animal Genetics and Breeding of the Ministry of Agricultural, Beijing Key Laboratory for Animal Genetic Improvement, College of Animal Science and Technology, China Agricultural University, Beijing 100193, China; jipengyun@cau.edu.cn (P.J.); gaoyu0910@cau.edu.cn (Y.G.); luzhang2018@cau.edu.cn (L.Z.)

**Keywords:** heat stress, Hainan black goat, melatonin, metabolome, metagenome, microbiota

## Abstract

As the global climate changes, high temperatures will cause heat stress, which significantly affects the productive efficiency of livestock. Currently, there is a lack of efficient methods to use in targeting this issue. In this study, we report that melatonin supplementation may represent an alternative method to reduce the negative impact of heat stress on livestock, particularly in Hainan black goats. Our results show that melatonin treatment increased the average daily gain of Hainan black goats that were exposed to constantly high temperatures for two months compared to controls. Our mechanistic exploration revealed that melatonin treatment not only reduced the oxidative stress and inflammatory reaction caused by heat stress but also improved goats’ metabolic capacity, promoting their growth and development. More importantly, for the first time, we observed that melatonin treatment modified the abundance of the intestinal microflora, altering the metabolism of the goats, which further improved their tolerance to constant heat stress.

## 1. Introduction

Heat stress in livestock is a frequently encountered and serious condition characterized by an imbalance between heat production and heat dissipation, often due to an environmental high temperature [1]. Heat stress usually leads to livestock having a reduced food intake, growth retardation, intestinal disorders, reproductive impairment, immunity deficiency, and endocrine system disability, all of which can cause productive deficiency in livestock [2,3]. For example, when the temperature is ≥32 °C, estrus and embryo survival rates are reduced in ewes and the fertilization rate is lower in rams [4]. Heat stress can also prolong the time needed for placental release in Saanen dairy goats, reduce the weight of placental cotyledons, and increase lamb mortality [5].

The Hainan black goat is a subspecies of black goat that is widely distributed in the Hainan Province of South China [6]. This area is part of the tropical maritime climate zone, with high temperatures and heavy rainfall throughout the year. The average annual temperature ranges from 25 °C to 28 °C, and during the summer, the temperature often exceeds 35 °C. The Hainan black goat has shown good tolerance to this climate and tolerates the high temperatures well compared to other breeds; however, the heat stress caused by these temperatures still negatively impacts their growth and productivity. To further improve the adaptation of Hainan black goats to the high temperatures in their habitat, we selected melatonin as a potential nutraceutical molecule to boost the tolerance of this species to heat stress.

Melatonin is mainly secreted by the pineal gland in mammals and has a variety of biological functions, including the regulation of the biological rhythm, antioxidative activity, immunity enhancement regulation, and retarding aging, as well as relieving heat stress in livestock [7,8]. Research has found that melatonin enhances the antioxidant capacity to reduce malondialdehyde (MDA) and nitric oxide (NO) levels and increases the estrus rate to 90% in water buffalo under heat stress conditions [9]. It improves the redox status of ewes under heat stress, increasing their average litter size and milk yield [10]. Additional studies have shown that melatonin supplementation alleviates testicular damage caused by heat stress in dairy goats by inhibiting the PI3K/AKT signaling pathway to reduce p-AKT levels and promoting BCL-2 levels [11] and also reduces DNA damage and apoptosis by inhibiting mitochondrial reactive oxygen species (ROS) production, increasing mitochondrial membrane potential, and reducing the formation of the lipid peroxidation product of 4-HNE in human sperm [12].

It is widely known that metabolic disorder is a typical feature of livestock under heat stress [13]. Animals subjected to long-term heat stress have reduced muscle glycogen storage, resulting in dark, hard, dry meat with a high pH [14]. Heat stress also causes gut microbiota dysregulation in bulls, resulting in energy metabolic imbalance and reduced fertility [15]. A growing body of research suggests that melatonin has a significant impact on the gut microbiome, which can improve gut health by reducing oxidative stress, autophagy, and inflammation [16,17]. Additionally, melatonin inhibits harmful Acinetobacter and promotes beneficial bacteria in the rumen flora [18].

Based on the available evidence, we hypothesized that melatonin would improve the tolerance of the Hainan black goat to heat stress, improving their productivity. Therefore, in this study, we systemically explored the effects of melatonin on heat stress, as well as the underlying molecular mechanisms, in Hainan black goats. Since metabolomics enables the simultaneous qualitative and quantitative analysis of all low-molecular-weight metabolites in an organism or cell during a specific physiological period [19], this method is also utilized in this study.

## 2. Materials and Methods

All the treatment protocols involving experimental animals in this study were approved by the Animal Welfare Committee of China Agricultural University; the approval protocol number is AW13014202-1-9.

### 2.1. Hainan Black Goat Breeding Management and Sample Collection

The Hainan black goats (*Capra hircus*) were raised at the Sanya Yazhou Beiling black goat farmers’ professional cooperative. The 45 experimental goats (weighing about 20 kg at three months old) were exposed to 27 °C~33 °C with 70%~89% humidity with a local natural light/dark cycle to generate heat stress. The goats were divided into 3 groups, each with 15 goats: a control group and groups treated with two different melatonin doses (1.5 and 4 mg/kg). The melatonin was purchased from Huanggang Hengxingyuan Chemical Co., Ltd., Huanggang, China, the production batch number was 230,220, and the purity was ≥99.5%. All experimental goats were fed twice daily, at 8:30 AM and 5:00 PM, with free access to water; melatonin was evenly mixed into the diet for feeding at 8:30 AM. The feed brand was “Yang Guanjia Meat goat Concentrated Feed”, and the production batch number was YN903-F40. All goats were housed in the same facility, and the environment was monitored by a temperature and humidity meter (JHT-2). The feeding start date was defined as Day 1. On Day 1, 15, 30, 45, and 60 of the study, body weights and body measurements were recorded (N = 15, each group). The serum was collected on Day 1, 30, and 60 and stored at −20 °C (N = 6, each group). Rectal fecal samples were collected on Day 30, under sterile conditions (N = 6, each group), flash-frozen in liquid nitrogen, and stored at −80 °C.

### 2.2. Measurements of Serum Hormone and Biochemical Indexes

(1) Hormone measurement: Cortisol [RXJ1100330G-96T], adrenocorticotropic hormone (ACTH) [RXJ1100152G-96T], and growth hormone (GH) [RX1100362G-96T] were detected by ELISA. The specific steps were as follows: ① All types of reagent were balanced at room temperature for two hours, and high-concentration washing liquid was diluted with distilled water at 1:20, according to the quantity of the batch, and mixed for reserve use. ② The pre-packed plate was taken out of the sealed bag, and a blank control hole was set up, with no liquid added; each calibrator was provided with 2 holes, each hole was added to the corresponding calibrator, and then 50 μL of serum or quality control product was added to every other test hole. ③ Biotinylated antigen (50 μL) was added to the cortisol and adrenocorticotropin detection hole, horseradish peroxidase (HRP)-labeled detection antibody (100 μL) was added to the growth hormone detection hole, and nothing was added to the blank hole. The sealing film was affixed, followed by incubation at 37 °C for 60 min. ④ The liquid in the holes was discarded, each hole was filled with the washing solution, and the cortisol and adrenocorticotropin test samples were left for 10 s and then shaken dry; this was repeated 3 times, and then they were patted dry. The growth hormone test hole was left to stand for 20 s, then the washing solution was shaken off and it was patted dry on absorbent paper; this process was repeated 5 times. ⑤ To the cortisol and adrenocorticotropin test holes (except for the blank control holes), 50 μL was added, and then the solution was mixed well, pasted with plate-sealing film, and incubated at 37 °C for 30 min. The liquid was discarded from the hole, and then each hole was filled with the washing liquid, left for 10 s, and shaken dry. This was repeated 3 times, and then each hole was patted dry. ⑥ Color-developing agents A and B (50 μL each) were added to the detection hole, and then the solution was shaken and mixed well and left at 37 °C away from light for 15 min before 50 μL of termination solution was added to each hole. ⑦ Readings were taken with an enzyme spectrometer; a wavelength of 450 nm was used, the zero point with the blank control hole was adjusted, and then the optical density value (OD value) was determined for each hole. After detection had been performed, the standard product concentration was taken as the ordinate coordinate, and the corresponding absorbance (OD value) was taken as the abscissa coordinate. Using computer software, the standard curve equation was created using four-parameter logistic curve fitting (4-pl). Through absorbance (OD value), the concentration value of the sample was calculated using the equation.

(2) Biochemical index measurements: Heat shock protein 70 (HSP-70) [RX1100310G], diamine oxidase (DAO) [RX1100419G-96T], lipopolysaccharide (LPS) [RXJ1100351G-96T], and D-lactic acid (D-LA) [RXJ1100418G-96T] were detected by ELISA. The operation method for heat shock protein 70 (HSP-70) and diamine oxidase (DAO) was consistent with the detection method for growth hormone. The testing method for lipopolysaccharide (LPS) [RXJ1100351G-96T] and D-lactic acid (D-LA) [RXJ1100418G-96T] were consistent with that used for adrenocorticotropic hormone.

(3) Inflammatory factor measurements: Tumor necrosis factor-α (TNF-α) [RX1100349G-96T] and Interleukin-1β (IL-1β) [RX1100289G-96T] were detected via ELISA. The testing method was consistent with the detection method used for growth hormones. The above testing work was entrusted to Quanzhou Ruixin Biotechnology Co., Ltd., Quanzhou, China.

(4) Antioxidant factor measurements: The total antioxidant capacity (T-AOC) was detected using the [A015-1-2] kit. The principle behind this kit is that the antioxidants in the body can reduce Fe^3+^ to Fe^2+^, which can form a stable complex with phenolines. The absorbance of each tube was measured at the wavelength of 520 nm using an enzyme labeler, and the activity of (T-AOC) in the measured sample was calculated using the formula. Superoxide dismutase (SOD) was detected using the [A001-1-2] kit. The kit principle is that the xanthine and xanthine oxidase reaction system produces superoxide anion radicals and the latter oxidizes hydroxylamine to form nitrite. The absorbance was measured at 550 nm using a visible-light spectrophotometer under the action of a color developer. When the sample contained SOD, the superoxide anion radical had a specific inhibition effect so that the formation of nitrite was reduced and the absorbance value of the measurement tube was lower than that of the control. The SOD activity in the measured sample was calculated via the formula. MDA was detected using the [A003-1-2] kit. Malondialdehyde (MDA) can be condensed with thiobarbituric acid (TBA) to form a red product with a maximum absorption peak of 532 nm. The absorbance was measured using an enzymoleter, and the MDA content was calculated via the formula. Catalase (CAT) was detected using the [A007-1-1] kit. The catalase decomposition of H_2_O_2_ can be quickly halted by adding ammonium molybdate. The remaining H_2_O_2_ reacts with ammonium molybdate to produce a yellowish complex. The activity of CAT can be calculated by measuring the change in CAT at 405 nm with an enzymoleter. The above testing work was entrusted to Nanjing Jiancheng Bioengineering Research Institute Co., Ltd., Nanjing, China.

(5) Fatty acid determination: The determination of acetic acid, isobutyric acid, butyric acid, isovaleric acid, propionic acid, valeric acid, and caproic acid was performed by Shanghai Meiji Biomedical Technology Co., Ltd., Shanghai, China. A certain quality of fecal sample was accurately weighed, and the extraction solution was added at a low temperature for metabolite extraction. The standard solution with different concentrations was prepared, and the standard solution and the sample were tested using the 8890B-5977B GC/MSD from Agilent Technologies Inc. (USA) under the same conditions. The default parameters of Masshunter quantitative software (Agilent, Santa Clara, CA, USA, version: v10.0.707.0) were used to automatically identify and integrate the ion fragments of target short-chain fatty acids. The actual content of short-chain fatty acids in samples was obtained by calculating the concentration of each sample through the standard curve.

### 2.3. The Serum Metabolome Analysis

Sample preparation: We mixed 100 μL of goat serum sample with 400 μL of extraction solution (acetonitrile–methanol = 1:1) containing an internal standard of 0.02 mg/mL (L-2-chlorophenylalanine) in a 1.5 mL centrifuge tube, which was vortexed for 30 s and then sonicated under a low temperature for 30 min (5 °C, 40KHz). The samples were left at −20 °C for 30 min, then centrifuged at 13,000× *g* for 15 min at 4 °C. The supernatant was collected and dried under nitrogen gas, then it was reconstituted with 100 μL of reconstitution solution (acetonitrile–water = 1:1), and low-temperature ultrasonic extraction was performed for 5 min (5 °C, 40 KHz) with centrifugation at 13,000× *g* for 10 min at 4 °C. The supernatant was placed into an autosampler vial for analysis. Quality control (QC) of the samples: Metabolites of all samples of equal volume were mixed in preparation for QC. A QC sample was inserted every 5–15 samples during instrument analysis to assess the reproducibility of the process and provide more details on the measurements. LC-MS/MS analysis: An ultra-high-performance liquid chromatography–tandem Fourier transform mass spectrometry (UHPLC-Q Exactive HF-X, Thermo Scientific, Waltham, MA, USA) system was used to analyze the samples. The 3 μL samples were separated using an HSST3 column (100 mm × 2.1 mmi.d., 1.8 µm) and then detected by mass spectrometry. Mobile phase A consisted of 95% water + 5% acetonitrile (containing 0.1% formic acid) and mobile phase B consisted of 47.5% acetonitrile + 47.5% isopropyl alcohol + 5% water (containing 0.1% formic acid). The flow rate was 0.40 mL/min, and the column temperature was 40 °C. The sample quality spectrum signals were acquired using positive- and negative-ion scanning modes, with a mass scan range of 70–1050 *m*/*z*. The sheath gas flow rate was 50 psi, the auxiliary gas flow rate was 13 psi, and the auxiliary gas heating temperature was 425 °C. The spray voltage was set to 3500 V in positive-ion mode and −3500 V in negative-ion mode. The ion transfer tube temperature was 325 °C, and the normalized collision energy was set to 20-40-60. Bioinformatics analysis: Bioinformatics analysis was performed using the official Majorbio platform (https://www.majorbio.com/web/login/passport/login-email?redirect_url=https://cloud.majorbio.com/page/project/overview.html, accessed on 12 Marth 2024). In brief, the data matrix was processed using the 80% rule to handle missing values, retaining variables with non-zero values in at least 80% of samples in at least one group. Missing values were then filled in using the minimum value from the original matrix. To minimize errors caused by sample preparation and instrument instability, total sum normalization (TSN) was applied to normalize the mass spectrometry peak intensities, yielding a normalized data matrix. Variables with a relative standard deviation (RSD) > 30% in QC samples were removed, and the data matrix was log10-transformed for subsequent analysis. The preprocessed data matrix was then subjected to principal component analysis (PCA) and orthogonal partial least squares–discriminant analysis (OPLS-DA) using the ropls package (Version 1.6.2) in R. The stability of the model was evaluated using 7-fold cross-validation. Significant differential metabolites were identified based on the variable importance in projection (VIP) scores from the OPLS-DA model and the *p*-values from Student’s *t*-tests, with metabolites satisfying VIP > 1 and *p* < 0.05 considered significantly different.

### 2.4. Fecal Metagenome Sequencing and Analysis

DNA Extraction and Sequencing: Fecal DNA was extracted using the E.Z.N.A.^®^ Soil DNA Kit (Omega Bio-tek, Norcross, GA, USA). DNA concentration was measured with the TBS-380, and DNA purity was assessed using the NanoDrop 2000. The integrity of the DNA was checked using 1% agarose gel electrophoresis. The DNA was fragmented using the Covaris M220 (BGI, Shenzhen, China), and fragments around 100 bp were selected. PE libraries were constructed using the NEXTFLEX™ Rapid DNA-SeqKit library building kit (BiooScientific, Austin, TX, USA). After bridge PCR amplification, metagenomic sequencing was conducted using the Illumina NovaSeq/HiSeq Xten (Illumina, San Diego, CA, USA) platform. Data Quality Control and Assembly: Fastp software (Version 0.20.0) was used for the quality control of the raw data, and BWA software (Version 0.7.9a) was used to align reads to the host DNA sequence, while contaminated reads with high similarity were removed. MEGAHIT software, based on the succinct de Bruijn graph algorithm, was used to assemble the optimized sequences. Contigs longer than or equal to 300 bp were selected as the final assembly results. MetaGene was used to predict the assembled contig with open reading frame (ORF), and CD-HIT software (Version 4.6.1) was used to cluster the predicted gene sequences of all the samples to construct the non-redundant gene set. SOAPaligner (Version 2.21) was then used to align high-quality reads from each sample with the non-redundant gene set (with 95% identity) to calculate the gene abundance in each sample. Bioinformatics Analysis: Bioinformatics analysis was conducted using the official Majorbio platform (https://www.majorbio.com/web/login/passport/login-email?redirect_url=https://cloud.majorbio.com/page/project/overview.html, accessed on 12 Marth 2024).

### 2.5. Statistical Analyses

The data are expressed as means ± SEM. SPSS.20 statistical software (SPSS Inc., Chicago, IL, USA) was used for the statistical analysis of body size data and blood biochemical indexes in Hainan black goats. If the data met the homogeneity test of variance, one-way ANOVA was used; if the data did not meet the homogeneity test of variance, a non-parametric test was used. The Kruskal–Wallis rank-sum test, one-way ANOVA, the Wilcoxon rank-sum test, and Student’s *t*-test were used to identify the differences between groups depending on the nature of each sample. *p* < 0.05 was considered statistically significant.

## 3. Results

The present study reports the effects of melatonin on the growth and development of Hainan black goats under heat stress. Melatonin decreased the oxidative stress and inflammatory response caused by heat stress in Hainan black goats and changed the abundance of the intestinal flora, thus changing the metabolism of goats and increasing the average daily weight gain in Hainan black goats.

### 3.1. Effects of Melatonin on the Growth of Hainan Black Goats Under Heat Stress

**Figure 1:**
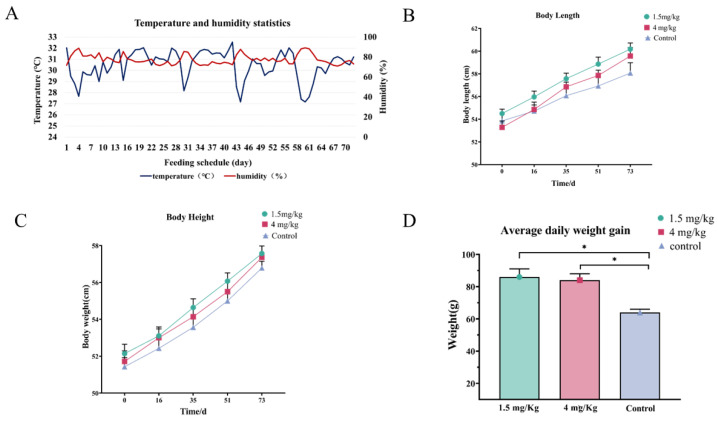
Body size data for lambs fed melatonin under heat stress. (**A**) Temperature and humidity statistics. (**B**) Body length. (**C**) Body height. (**D**) Average daily weight gain. * *p* < 0.01. The data are expressed as mean ± SEM, N = 15. * *p* < 0.05 between compared groups.

In this study, the temperature and humidity of the goat barn environment were monitored. As shown in Figure 1A, the temperature and humidity of the barn ranged from 27 °C to 33 °C and 70% to 89%, respectively, indicating that the goats were constantly exposed to heat stress. In terms of the severity of heat stress, over a 24-h period, severe heat stress lasted for 2.8 h, while moderate heat stress persisted for 21 h. The results showed that under the heat stress caused by such high temperatures and humidity, the average daily weight gain in goats treated with both doses of melatonin significantly increased compared to the control group (86.00 ± 5.01 or 84.00 ± 4.00 vs. 4.00 ± 2.01 g) (Figure 1D). The body length and body height were also increased in the melatonin-treated group, but the difference was not significant compared to the control (*p* > 0.05) (Figure 1B,C).

### 3.2. Effects of Melatonin on Blood Biochemical Indexes and Short-Chain Fatty Acids in Hainan Black Goats Under Heat Stress

**Figure 2:**
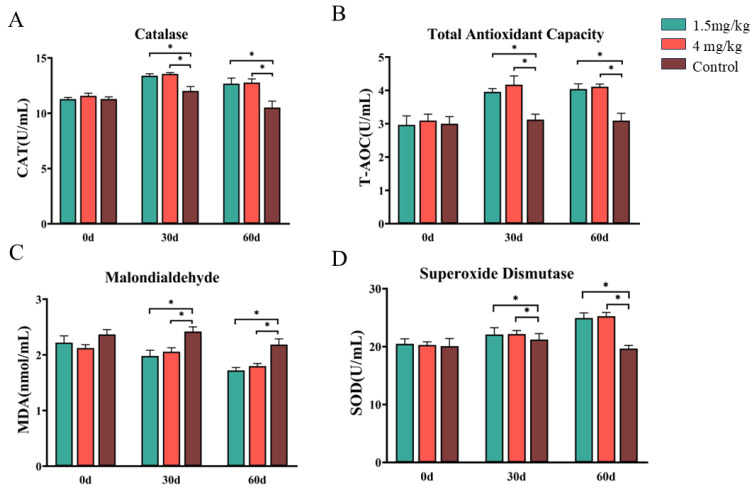
Effects of melatonin on antioxidant factors in Hainan black goats. (**A**) Catalase content. (**B**) Total antioxidant capacity. (**C**) Malondialdehyde content. (**D**) Superoxide dismutase content. The data are expressed as mean ± SEM, N = 6. * *p* < 0.05 between compared groups.

**Figure 3:**
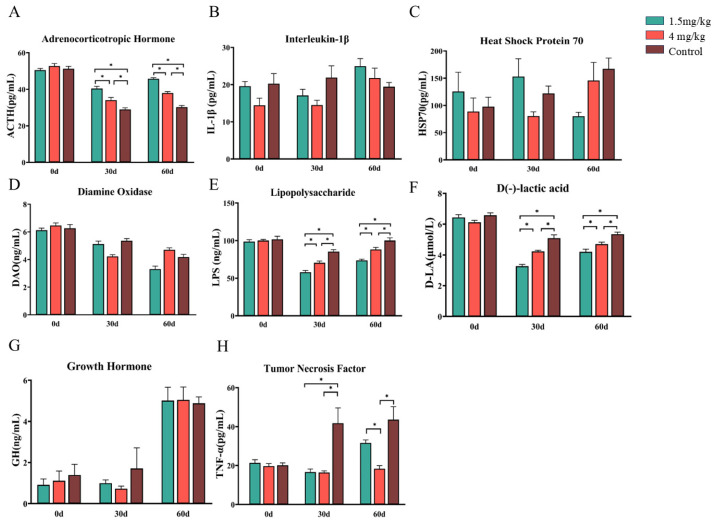
Effects of melatonin on blood biochemical indexes in Hainan black goats under heat stress. (**A**) Adrenocorticotropin. (**B**) Heat shock protein-70. (**C**) Diamine oxidase. (**D**) Endotoxin. (**E**) D-lactic acid. (**F**) Growth hormone. (**G**) Tumor necrosis factor-α. (**H**) Interleukin-1β. The data are expressed as mean ± SEM, N = 6. * *p* < 0.05 between compared groups.

**Figure 4:**
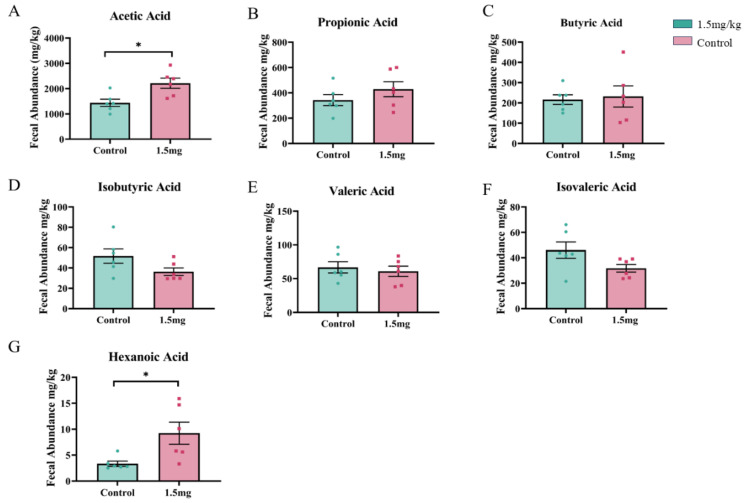
Effect of melatonin on short-chain fatty acid concentration in Hainan black goat feces under heat stress. (**A**) Acetic acid, (**B**) propionic acid, (**C**) butyric acid, (**D**) isobutyric acid, (**E**) isovaleric acid, (**F**) valeric acid, (**G**) caproic acid. *p* < 0.05. The data are expressed as mean ± SEM, N = 6, * *p* < 0.05 between compared groups.

An analysis of blood antioxidant capacity showed that both doses of melatonin treatment significantly increased the total antioxidant capacity and the activities of catalase and superoxide dismutase while significantly reducing the content of malondialdehyde compared to the control group (*p* < 0.05). These parameters were not significantly different between the two melatonin groups (*p* > 0.05) (Figure 2A–D). Melatonin treatment also significantly increased adrenocorticotropic hormone levels compared to the control group (*p* < 0.05), with a better effect in the 1.5 mg melatonin group than in the 4 mg melatonin group (Figure 3A). On the other hand, melatonin treatment significantly lowered lipopolysaccharide, D (-)-lactic acid, and tumor necrosis factor-α levels compared to the control group, with better effects seen in terms of lowering lipopolysaccharide and D (-)-lactic acid in the 1.5 mg melatonin-treated group than in the 4 mg melatonin-treated group (Figure 3D,E) (*p* < 0.05). Meanwhile, on Day 60, the tumor necrosis factor-α level in the 1.5 mg melatonin group was significantly higher than in the 4 mg melatonin group (Figure 3G) (*p* < 0.05). Melatonin treatment had no significant effect on heat shock protein, growth hormone, diamine oxidase, or Interleukin-1β levels compared to the control group (*p* > 0.05). Additionally, melatonin treatment significantly increased the concentrations of acetic acid and caproic acid in feces (*p* < 0.05) but had no significant effect on other short-chain fatty acids compared to the control group (*p* < 0.05) (Figure 4A,G).

### 3.3. Effects of Melatonin on Non-Targeted Metabolome Differential Metabolites in Hainan Black Goats Under Heat Stress

**Figure 5:**
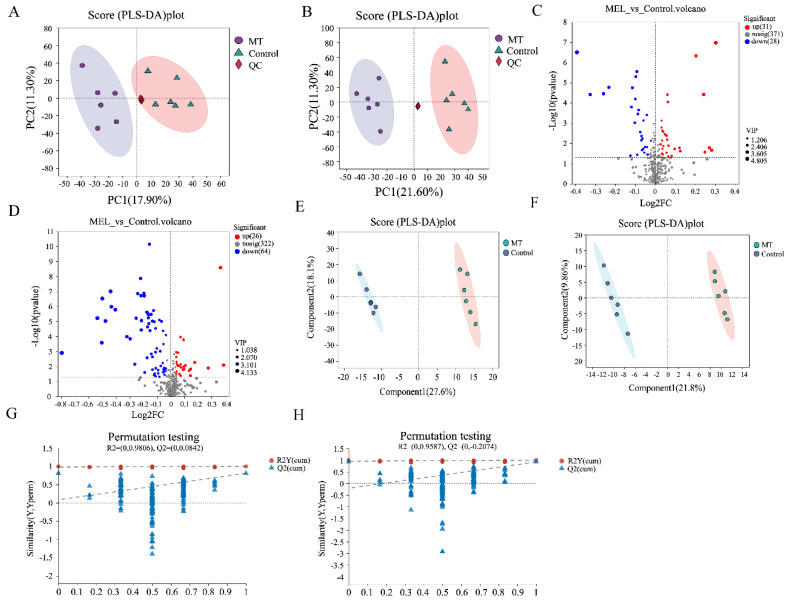
Effects of melatonin on non-targeted metabolome differential metabolites in Hainan black goats under heat stress. (**A**) Principal component analysis (fecal cationic model). (**B**) Principal component analysis (fecal anion model). (**C**) Volcanic maps of differentiated metabolites between groups (fecal positive-ion model). (**D**) Volcanic maps of differentiated metabolites between groups (fecal anion model). (**E**) Verification of the robustness of OPLS-DA model in forward mode based on 200 permutation tests. (**F**) Verification of the robustness of OPLS-DA model in negative mode based on 200 permutation tests. (**G**) Score chart of OPLS-DA in forward mode. (**H**) Score chart of OPLS-DA in negative mode.

Non-targeted metabolomics analysis was performed on blood from the different groups of Hainan black goats. The results showed that in positive-ion mode, there were 3408 effective peaks (raw), 3243 pre-treated effective peaks (origin), and 430 identified metabolites (origin). In the negative-ion mode, there were 4024 effective peaks (raw), 3870 pre-treatment effective peaks (origin), and 409 identified metabolites (origin). As shown in Figure 5A,B, QC samples were clustered and clearly separated from test serum samples in the melatonin-treated and control groups, indicating high-quality biological analysis and data. A total of 119 different metabolites between the groups were screened: 31 metabolites were upregulated and 28 metabolites were downregulated in positive-ion mode (Figure 5C), while 26 metabolites were upregulated and 64 were downregulated in negative-ion mode (Figure 5D). PLS-DA analysis showed that, for cations, Component 1 explained 21.8% of the variance and Component 2 explained 9.86%. The PLS-DA permutation test yielded R^2^ = 0.9806 and Q^2^ = 0.0842. For anions, Component 1 explained 27.6% of the variance and Component 2 explained 18.1%. The PLS-DA permutation test yielded R^2^ = 0.9587 and Q^2^ = −0.2074. The R^2^ values for both the positive- and negative-ion modes exceeded 0.9, but the Q^2^ values were low, indicating that there were significant metabolic differences between the melatonin-treated and control groups under heat stress (Figure 5G,H).

### 3.4. Analysis of Differentially Functional Pathways of Non-Targeted Metabolites Between Melatonin-Treated and Control Groups

**Figure 6:**
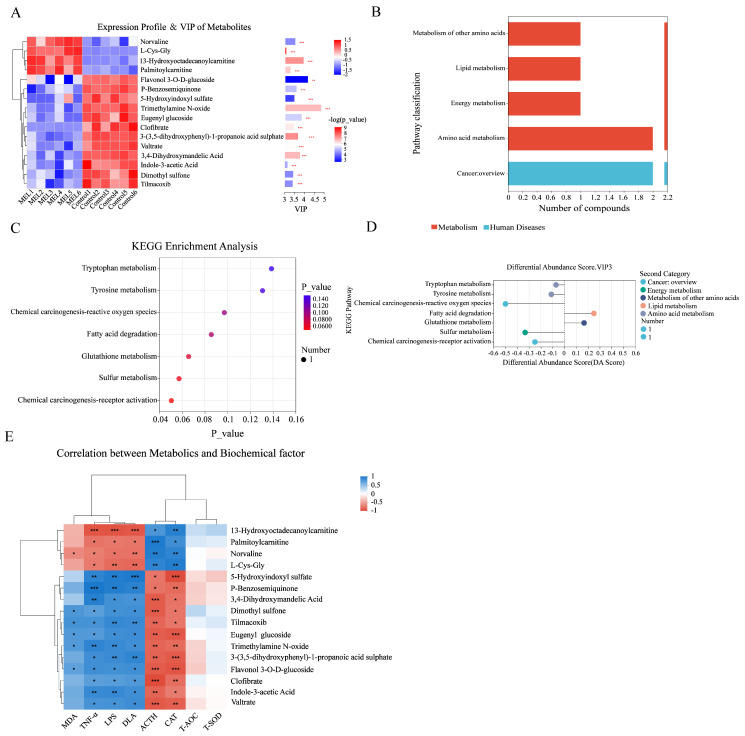
Analysis of differential metabolite functional pathways among non-targeted metabolites between melatonin-treated and control groups. (**A**) VIP analysis. (**B**) KEGG statistical chart. (**C**) KEGG enrichment analysis diagram. (**D**) KEGG channel differential abundance score map. (**E**) Correlation heat map. * *p* < 0.05, ** *p* < 0.01, and *** *p* < 0.001.

Following the requirement of VIP > 3 in PLS-DA analysis, FC ≥ |1.1|, univariate analysis *p* < 0.01, and FDR < 0.05 to screen for differentiated metabolites between groups, 16 significantly differentiated metabolites were identified. Among them, 4 metabolites were significantly upregulated and 12 were significantly downregulated, showing significant differences between the melatonin-treated and control groups (Figure 6A). KEGG functional pathway statistics showed that the differential metabolites were mainly concentrated on metabolic pathways (Figure 6B). KEGG enrichment analysis showed that the most significant pathway was “Chemical carcinogenesis-receptor activation”, with overall downregulation (Figure 6C,D), while the tryptophan metabolism pathway was not significantly influenced. Correlation analysis was performed between the 16 differential metabolites and the significantly different antioxidant factors in serum (catalase, total antioxidant capacity, superoxide dismutase, and malondialdehyde) as well as four biochemical markers (adrenocorticotropic hormone, endotoxin, D-lactic acid, and tumor necrosis factor-α). The results showed that these 16 metabolites were significantly associated with adrenocorticotropin, endotoxin, D-lactic acid, tumor necrosis factor-α, and catalase. Norvaline was significantly correlated with Dimethyl-sulfone, Tilmacoxib, Eugenylglucoside, TrimethylamineN-oxide, Flavonol3-O-D-glucoside, and MDA (Figure 6E).

### 3.5. Effects of Melatonin Treatment on Metagenomic Microbial Flora Under Heat Stress

**Figure 7:**
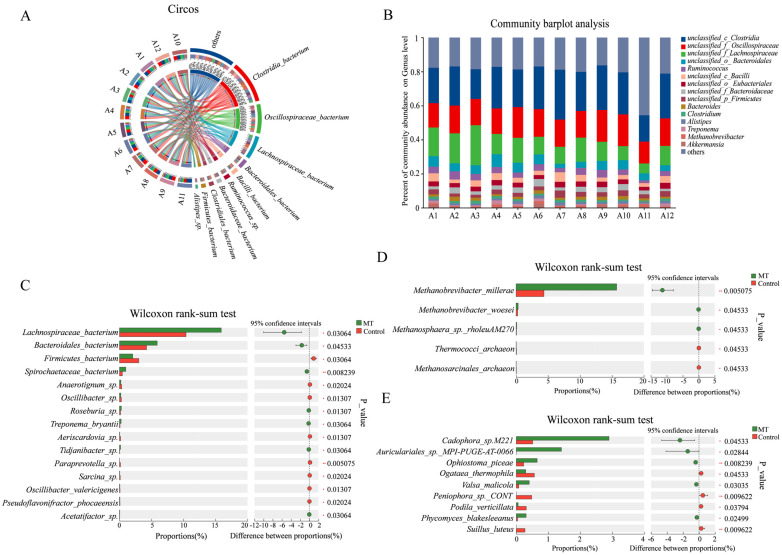
The results of microbiota analysis on samples from the melatonin-treated and control groups of Hainan black goats under heat stress. (**A**) Circos map of the microbial community. (**B**) Genus horizontal community histogram. (**C**) Histogram of Wilcoxon rank-sum test for d-Bacteria levels. (**D**) Histogram of k-Fungi-level Wilcoxon rank-sum test. (**E**) d-Archaea horizontal Wilcoxon rank-sum test histogram.

A total of 12 samples from the control group and melatonin group (1.5 mg/kg) were selected for metagenomic analysis. After quality control and data processing, an average of 75,042,545.5 clean reads and 11,249,765,288 bases were obtained. The clean reads accounted for more than 98.2% of the raw reads, and the clean bases accounted for 97.5% of the raw bases. This indicated that the sequencing data were of good quality and suitable for subsequent analysis. Species annotation identified 14, 209, 387, 767, 1517, 4173, and 16,758 species at the kingdom (K), phylum (P), class (C), order (O), family (F), genus (G), and species (S) taxonomic levels, respectively. The horizontal distribution of the microbial species and genus is shown in Figure 7A,B. The fecal metagenomic data consisted of 97.31% bacteria, 1.16% archaea, 0.92% eukaryotes, 0.59% viruses, and 0.001% unclassified species. After Wilcoxon rank-sum test analysis, the main bacterial differences between groups were found in *Lachnospiraceae bacterium* and *Bacteroidales bacterium.* The fungal differences were mainly concentrated in *Cadophora* sp. *M221* and *Auriculariales* sp. *MPI-PUGE-AT-0066*, while archaeal differences were mainly found in *Methanobrevibacter millerae* and *Methanobrevibacter woesei*.

### 3.6. The Results of Wilcoxon Rank-Sum Test Analysis of Different Databases

**Figure 8:**
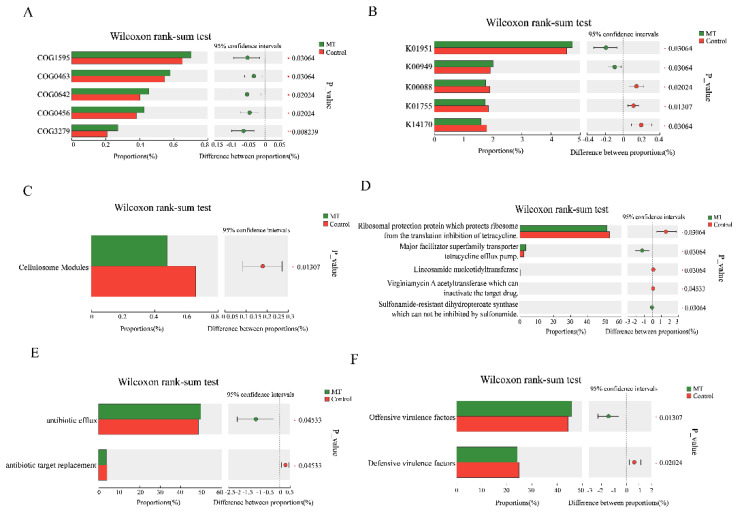
Results of Wilcoxon rank-sum test analysis for different databases. (**A**) COG database. (**B**) KEGG database. (**C**) CAZy database. (**D**) ARDB database. (**E**) CARD database. (**F**) VFDB database.

The Wilcoxon rank-sum test was performed to analyze the microflora of the two groups. Among the COG databases, the most significant difference was seen for COG1595 (Figure 8A). The most significant difference in the KEGG database was noted for K01951 (Figure 8B). The most significant difference between the CAZy databases was seen for Cellulosome Modules (Figure 8C). The most significantly different pathway in the ARDB database was that of Ribosomal protection protein, which protects ribosomes from the translation inhibition of tetracycline (Figure 8D). The most significant difference in the CARD database was in antibiotic efflux (Figure 8E). The most significant difference in the VFDB database was in the offensive virulence factors (Figure 8F).

### 3.7. Correlation Analysis of Intestinal Flora

**Figure 9:**
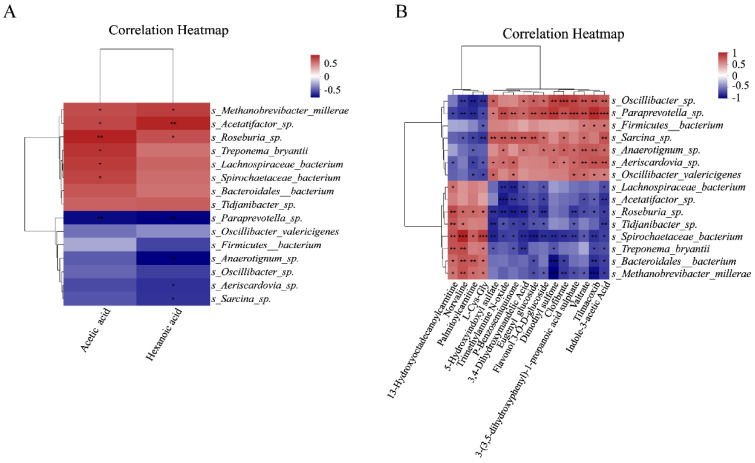
Correlation analysis of the intestinal microbial flora. (**A**) Correlation analysis of the microbial flora with acetic acid and caproic acid. (**B**) Association analysis between the microbial flora and metabolic differentials. * *p* < 0.05, ** *p* < 0.01, and *** *p* < 0.001.

At the species level, the top 15 species with the highest flora abundance between the melatonin-treated and control groups were selected, and the correlation of these species with acetic acid and caproic acid levels was analyzed. *Lachnospiraceae bacterium*, *Spirochaetaceae bacterium*, *Roseburia* sp., *Treponema bryantii*, *Methanobrevibacter millerae*, and *Acetatifactor* sp. were positively correlated with acetate levels in feces, while *Paraprevotella* sp. was negatively correlated with acetate levels. *Roseburia* sp., *Methanobrevibacter millerae*, and *Acetatifactor* sp. were positively correlated with hexanoic acid levels in feces, while *Anaerotignum* sp., *Aeriscardovia* sp., *Paraprevotella* sp., and *Sarcina* sp. were negatively correlated with hexanoic acid levels (Figure 9A) (*p* < 0.05). Then, the correlation between the different flora and the metabolites with significant differences was analyzed. Among the microbial taxa, *S. paraprevotella sp* and *S. Spirochaetaceae bacterium* were significantly correlated with all metabolites. *S. paraprevotella* sp. was significantly correlated with dimethylsulfone, 3-(3,5-dihydroxyphenyl)-1-propanoic acid sulfate, tilmacoxib, and indole-3-acetic acid. *S. Spirochaetaceae bacterium* was significantly correlated with norvaline, L-Cys-Gly, and eugenylglucoside. *S. Oscillibacter* sp. was significantly correlated with clofibrate, and *S. Acetatifactor* sp. was significantly correlated with trimethylamine-N-oxide. Dimethylsulfone was significantly correlated with *S. Bacteroidales bacterium* and *S. Methanobrevibacter millerae*, while tilmacoxib was significantly correlated with *S. Methanobrevibacter millerae* (Figure 9B) (*p* < 0.05).

## 4. Discussion

Due to the global greenhouse effect, the negative impact of heat stress on livestock growth is being dramatically increased [20]. Under heat stress, domestic animals show typical behaviors of listlessness and reduced feed intake, accompanied by metabolic imbalance and alterations in blood hormone and protein levels, as well as in antioxidant capacity [21,22]. Commonly used practical methods to mitigate the losses caused by heat stress include nutritional adjustments and environmental control, but the results have not been optimal. This has led researchers to continuously search for alternative methods to further lower the negative impact of heat stress on livestock.

Melatonin, a neuroendocrine hormone widely present in organisms, possesses strong antioxidant properties [23]. Numerous studies have shown that melatonin can reduce the damage caused by heat stress in livestock and improve farming efficiency [24]. However, it has not yet been used in Hainan black goats, which are widely distributed in Hainan Province, China, and are persistently exposed to environmental high temperatures and humidity. The Hainan Province of China is located in a tropical region with persistently high temperatures year round. Heat stress is an important factor that limits optimal production from animal husbandry in this region [25]. In this study, we systematically investigated the potential effects of melatonin on heat stress in Hainan block goats and also explored the underlying mechanisms that are mediated by this molecule.

During this study, Hainan black goats were constantly exposed to high temperatures and humidity to manifest heat stress and, at the same time, different doses of melatonin were continuously supplied to these animals for two months. During the study period, the blood biochemical indices, growth, and development of the Hainan black goats were closely monitored. The result showed that melatonin significantly increased the average daily body gain and the development of black goats under heat stress compared to the untreated control group. The results are consistent with previous reports on different species. For example, melatonin alleviated injury caused by heat stress in high-yield dairy cows [26] and protected the ovary structure and embryo quality of cows under heat stress by enhancing the activity of the SOD and lowering MDA levels [27]. Melatonin promotes lamb growth and development by mediating apoptosis signaling pathways and modifying the structure of the gut microbiota [28]. Accordingly, our results showed that melatonin treatment significantly increased the antioxidant capacity of Hainan black goats under heat stress, as indicated by the elevated activities of catalase and superoxide dismutase with a significant reduction in the malondialdehyde level. Choudhary et al. [29] have reported similar results to ours, which showed that melatonin significantly reduced malondialdehyde levels and increased the total antioxidant capacity and superoxide dismutase activity in Chhotanagpuri ewes. In non-human primates and rodents, melatonin acts directly on the adrenal glands and suppresses the action of adrenocorticotropic hormone (ACTH) [30]. However, in this study, melatonin significantly increased ACTH levels. This difference may be related to the species-specific or different experimental conditions, such as the fact that our animals were tested under heat stress conditions. This requires further investigation. Lipopolysaccharides [31], tumor necrosis factor-α (TNF-α) [32], and Interleukin-1β (IL-1β) [33] are important factors in immune system regulation and inflammation, and D(-)-lactic acid not only regulates protein metabolism and oxidative energy supply, but it also participates in modulating immune function [34]. In this study, melatonin reduced the expression levels of lipopolysaccharide, tumor necrosis factor-α, and D (-)-lactic acid. These actions by melatonin significantly suppress the inflammatory response of goats under heat stress. Diamine oxidase can protect the intestinal mucosa and maintain intestinal health [35]. When the body is under heat stress, heat shock proteins are synthesized in large quantities to protect it [36]. In our study, we failed to detect elevated levels of diamine oxidase and heat shock proteins in melatonin-treated animals compared to control animals. This can be explained by the fact that Hainan black goats have adapted to their tropical environment and have already maximized their expression of these proteins. In primates, melatonin increases the expression/release of growth hormone in a dose- and time-dependent manner [37]. However, in this study, melatonin had no significant effect on growth hormones, which may be another species-specific feature.

To explore the mechanisms by which melatonin promotes the tolerance of Hainan black goats to heat stress and promotes their growth and development, a non-targeted metabolomic analysis of their blood metabolites was conducted. A total of 119 differentiated metabolites were identified between melatonin-treated and control goats, indicating a significant effect of melatonin in black goat metabolism. Among them, 16 differentiated metabolites were further selected, of which 4 were significantly upregulated and 12 were significantly downregulated by melatonin treatment. Cell maintenance through metabolic regulation is a necessary process in cell functioning [38]. The KEGG function of differentiated metabolites confirmed that under heat stress, melatonin activity was mainly concentrated in metabolic pathways, which was consistent with a previous report [39]. The most notable effect of melatonin was observed in the chemical carcinogenesis-receptor activation pathway, and the overall expression of this pathway under heat stress was downregulated by melatonin treatment. Surprisingly, tryptophan metabolism was not affected by melatonin treatment, even though tryptophan is the precursor to melatonin biosynthesis [40]. To further explore the underlying mechanism behind the role of melatonin in heat stress in Hainan black goats, a correlation analysis was performed between these melatonin-affected biochemical parameters and the 16 significantly differentiated metabolites. The results showed that the 16 metabolites were significantly correlated with adrenocorticotropic hormone, endotoxins, d-lactic acid, tumor necrosis factor-α, and catalase. The results suggest that melatonin may influence metabolic pathways, which, in turn, affect physiological and biochemical factors in Hainan black goats.

More and more studies have shown that the gut microbiota not only affects nutrient absorption but also plays a key role in immune regulation. Under the stimulation of heat stress, the feed intake of livestock decreases, and this leads to alterations in the intestinal microflora. This dysbiosis, in turn, will aggravate heat stress syndrome [41]. Melatonin has beneficial effects on the distribution and metabolism of gut microorganisms [42,43]. This was confirmed in our study on Hainan black goats. The Wilcoxon rank-sum test showed that melatonin made differentiating changes in d-Bacteria, k-Fungi, and d-Archaea compared to the control goats. The differences in d-Archaea were mainly concentrated in *Methanobrevibacter millerae* and *Methanobrevibacter woesei*. This is consistent with previous studies showing that melatonin can reduce methanogens in the rumen of dairy cows [44]. When Wilcoxon rank-sum test analysis was applied to a variety of databases including COG and CAZy, both identified certain differences among groups of microorganisms. The correlation analysis found that six bacterial groups, including *Lachnospiraceae_bacterium*, were positively correlated with acetic acid levels and three bacterial groups, including *Roseburia_*sp., were positively correlated with caproic acid. The flora of *Paraprevotella_*sp. was negatively correlated with acetic acid levels, and the flora of *Anaerotignum_*sp. was negatively correlated with caproic acid. In addition, *S_paraprevotella_*sp. and *S_Spirochaetaceae_bacterium* were significantly correlated with all metabolites. Melatonin treatment significantly increased the fecal acetic acid and capric acid levels of Hainan black goats under heat stress conditions compared to the control goats. These findings suggest that the protective effects of melatonin in heat stress in Hainan black goats may also occur through alterations to the gut microbiota abundance, modifying their metabolic levels.

Many studies have reported the protective effects of melatonin in heat stress in livestock, including sheep [45]. However, this study is the first to report that melatonin promotes the growth rate of Hainan black goats in consistent (2 months) heat stress conditions. The results suggest that melatonin has broad prospects for application in the livestock industry, particularly in tropical areas with high temperatures and humidity. This study provided preliminary but useful information on the role of melatonin in promoting the growth and development of black goats or other livestock. Further research is necessary to learn more.

## 5. Conclusions

In this study, the protective effects of melatonin in heat stress in Hainan black goats were systemically investigated. The results showed that melatonin treatment increased the average daily gain of the goats compared to their controls. Mechanistic exploration using omics identified that melatonin not only reduced oxidative stress and the inflammatory reaction caused by heat stress in Hainan black goats but also, more importantly, modified the abundance of the intestinal microflora, altering the metabolism of the goats, and therefore further improving their tolerance to constant heat stress. This had never been reported previously.

## Data Availability

For raw data, please contact the corresponding author.
